# Selected PET radiomic features remain the same

**DOI:** 10.18632/oncotarget.25070

**Published:** 2018-04-17

**Authors:** Tetsuya Tsujikawa, Hideaki Tsuyoshi, Masafumi Kanno, Shizuka Yamada, Masato Kobayashi, Norihiko Narita, Hirohiko Kimura, Shigeharu Fujieda, Yoshio Yoshida, Hidehiko Okazawa

**Affiliations:** ^1^ Biomedical Imaging Research Center, University of Fukui, Fukui, Japan; ^2^ Department of Obstetrics and Gynecology, Faculty of Medical Sciences, University of Fukui, Fukui, Japan; ^3^ Department of Otolaryngology, Faculty of Medical Sciences, University of Fukui, Fukui, Japan; ^4^ Wellness Promotion Science Center, College of Medical, Pharmaceutical and Health Sciences, Kanazawa University, Kanazawa, Japan; ^5^ Department of Radiology, Faculty of Medical Sciences, University of Fukui, Fukui, Japan

**Keywords:** PET radiomic features, PET/CT, PET/MR

## Abstract

**Purpose:**

We investigated whether PET radiomic features are affected by differences in the scanner, scan protocol, and lesion location using ^18^F-FDG PET/CT and PET/MR scans.

**Results:**

SUV, TMR, skewness, kurtosis, entropy, and homogeneity strongly correlated between PET/CT and PET/MR images. SUVs were significantly higher on PET/MR_0-2 min_ and PET/MR_0-10 min_ than on PET/CT in gynecological cancer (*p* = 0.008 and 0.008, respectively), whereas no significant difference was observed between PET/CT, PET/MR_0–2 min_, and PET/MR_0–10 min_ images in oral cavity/oropharyngeal cancer. TMRs on PET/CT, PET/MR_0–2 min_, and PET/MR_0–10 min_ increased in this order in gynecological cancer and oral cavity/oropharyngeal cancer. In contrast to conventional and histogram indices, 4 textural features (entropy, homogeneity, SRE, and LRE) were not significantly different between PET/CT, PET/MR_0–2 min_, and PET/MR_0–10 min_ images.

**Conclusions:**

^18^F-FDG PET radiomic features strongly correlated between PET/CT and PET/MR images. Dixon-based attenuation correction on PET/MR images underestimated tumor tracer uptake more significantly in oral cavity/oropharyngeal cancer than in gynecological cancer. ^18^F-FDG PET textural features were affected less by differences in the scanner and scan protocol than conventional and histogram features, possibly due to the resampling process using a medium bin width.

**Methods:**

Eight patients with gynecological cancer and 7 with oral cavity/oropharyngeal cancer underwent a whole-body ^18^F-FDG PET/CT scan and regional PET/MR scan in one day. PET/MR scans were performed for 10 minutes in the list mode, and PET/CT and 0–2 min and 0–10 min PET/MR images were reconstructed. The standardized uptake value (SUV), tumor-to-muscle SUV ratio (TMR), skewness, kurtosis, entropy, homogeneity, short-run emphasis (SRE), and long-run emphasis (LRE) were compared between PET/CT, PET/MR_0-2 min_, and PET/MR_0-10 min_ images.

## INTRODUCTION

Recent positron emission tomography/computed tomography (PET/CT) systems have a time-of-flight (TOF) capability and point spread function (PSF) correction, which result in a high signal-to-noise ratio (SNR) and high spatial resolution [1]. PET/CT with 2-[^18^F]-fluoro-2-deoxy-D-glucose (^18^F-FDG) is of significant value in evaluations of cancer patients because it has the capacity to identify lymph node involvement, distant disease, and recurrence [2]. Radiomics is an emerging field that consists of the conversion of medical images into mineable data and subsequent analyses of these data for decision support [3, 4]. An ^18^F-FDG PET radiomic approach assesses global ^18^F-FDG uptake as well as locoregional heterogeneity in the distribution of ^18^F-FDG with textural feature measurements [5], which have been used to differentiate between malignant and benign lesions [6, 7], predict treatment responses and patient prognoses [8, 9], and characterize tumor phenotypes (histological and molecular subtypes) [10, 11].

The recent introduction of integrated PET and magnetic resonance (PET/MR) scanners has demonstrated the advantages of simultaneous PET and MR imaging with higher soft-tissue contrast, multiplanar image acquisition, functional imaging capability, and lower radiation exposure than PET/CT [12, 13]. One of the latest integrated PET/MR scanners, Signa PET/MR has silicon photomultipliers (SiPMs) with an excellent time resolution of <400 ps and enables TOF similar to PET/CT scanners [14]. Under existing conditions, ^18^F-FDG PET radiomic approaches may be performed using PET/CT and PET/MR scanners. However, differences exist in PET crystals, photomultipliers, and the attenuation correction (AC) of PET data between PET/CT and PET/MR scanners. The difference in AC is the greatest and most important for quantitation. Low-dose CT scans are now the standard for AC in whole-body PET/CT imaging. On the other hand, MR segmentation-based AC (MR-AC) is typically used in whole-body PET/MR imaging with the exception of CT atlas-based AC for the head. Since MR images do not reflect electron densities, the MR-AC map does not delineate bones or assign appropriate attenuation coefficients to them. MR-AC errors are considered to be prominent in gynecological cancer and oral cavity/oropharyngeal cancer due to the thick pelvic and jaw bones, respectively. These technological differences and biological factors may affect the quantification of PET radiomic features [15, 16].

The primary goals of the present study were two-fold. We investigated whether ^18^F-FDG PET radiomic features are affected by differences in the scanner, scan protocol, and lesion location using ^18^F-FDG PET/CT and PET/MR scans in patients with gynecological cancer and oral cavity/oropharyngeal cancer. We then aimed to suggest approapriate extraction methods and features to be used as quantitative measures in future multicenter studies. We herein adjusted reconstruction parameters between PET/CT and PET/MR images in order to reduce the impact of image reconstruction settings on PET radiomic features between scanners [17].

## RESULTS

### Relationships among PET radiomic features extracted using 64 bins (bin width = 0.4 SUV)

PET radiomic features extracted from 3 different PET images using 64 bins (fixed bin width of 0.4 SUV) in gynecological cancer and oral cavity/oropharyngeal cancer are shown in Table 1.

**Table 1:** PET radiomic features extracted using 64 bins (bin width = 0.4 SUV) from 3 different PET images

		Gynecological cancer						Oral cavity/oropharyngeal cancer					
		PET/CT		PET/MR_0–2 min_		PET/MR_0–10 min_		PET/CT		PET/MR_0–2 min_		PET/MR_0–10 min_	
Explanation	Feature	Mean	SD	Mean	SD	Mean	SD	Mean	SD	Mean	SD	Mean	SD
Conventional	SUV	8.9	3.3	11.5	4.7	11.2	4.5	11.0	3.7	11.1	3.3	10.9	3.1
	TMR	13.0	5.3	14.1	4.8	14.3	5.2	10.3	4.2	11.8	5.2	12.2	5.4
Histogram	Skewness	0.47	0.23	0.37	0.18	0.32	0.18	0.46	0.42	0.35	0.43	0.26	0.39
	Kurtosis	2.38	0.40	2.31	0.35	2.17	0.30	2.25	0.49	2.26	0.57	2.08	0.37
GLCM matrix	Entropy	2.74	0.31	2.82	0.30	2.80	0.36	2.55	0.31	2.42	0.36	2.40	0.38
	Homogeneity	0.23	0.08	0.22	0.07	0.24	0.09	0.19	0.03	0.18	0.02	0.18	0.02
GLRLM matrix	SRE	0.97	0.02	0.96	0.02	0.96	0.02	0.98	0.01	0.98	0.00	0.97	0.01
	LRE	1.16	0.09	1.18	0.10	1.20	0.13	1.10	0.02	1.08	0.02	1.08	0.02

**Figure 1:**
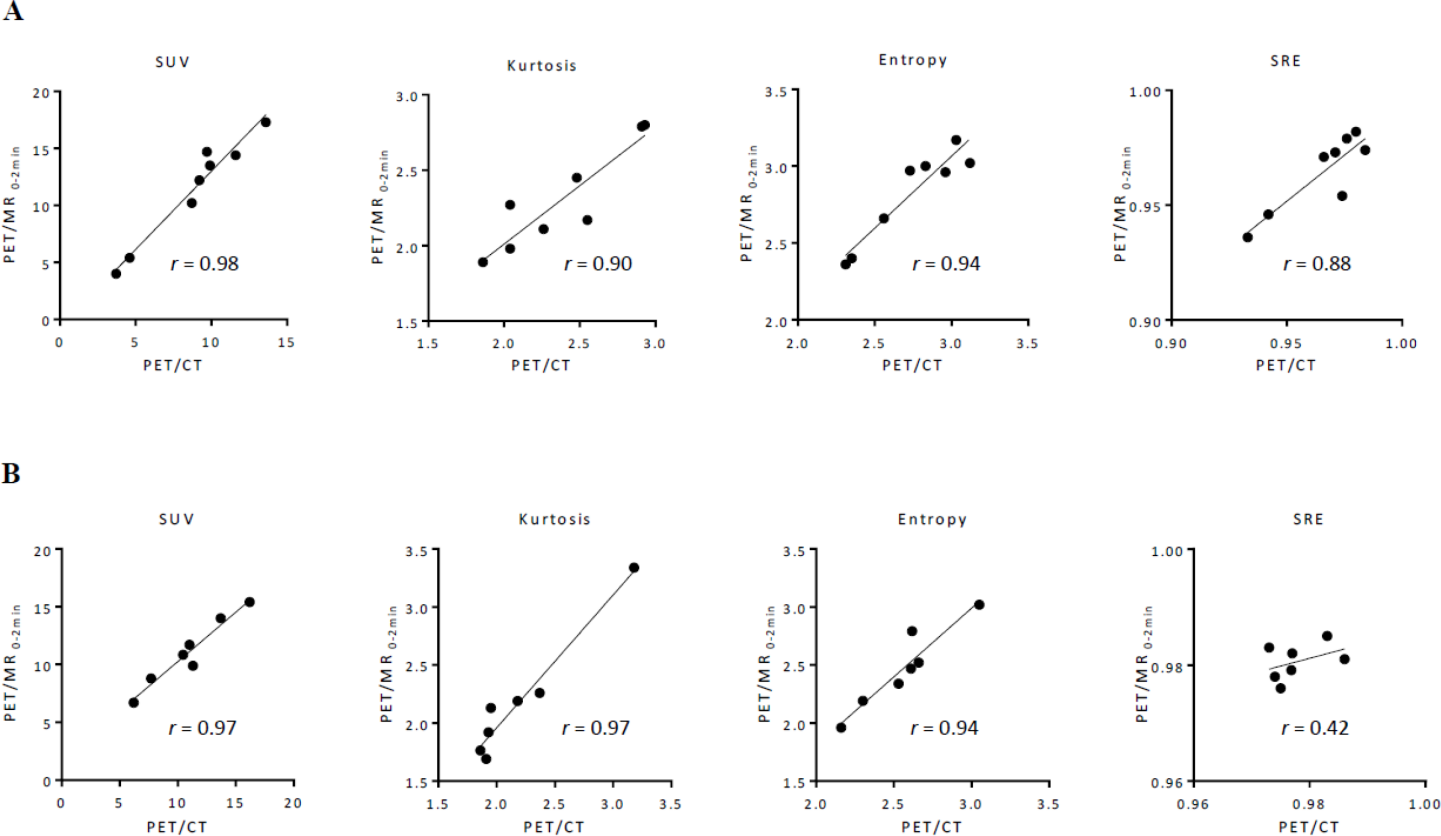
Relationships among features extracted using 64 bins (bin width = 0.4 SUV) between PET/CT and PET/MR_0–2 min_ images in gynecological cancer (**A**) and oral cavity/oropharyngeal cancer (**B**). A regression line is shown with Pearson’s correlation coefficient (*r*) and a *p* value.

**Figure 2:**
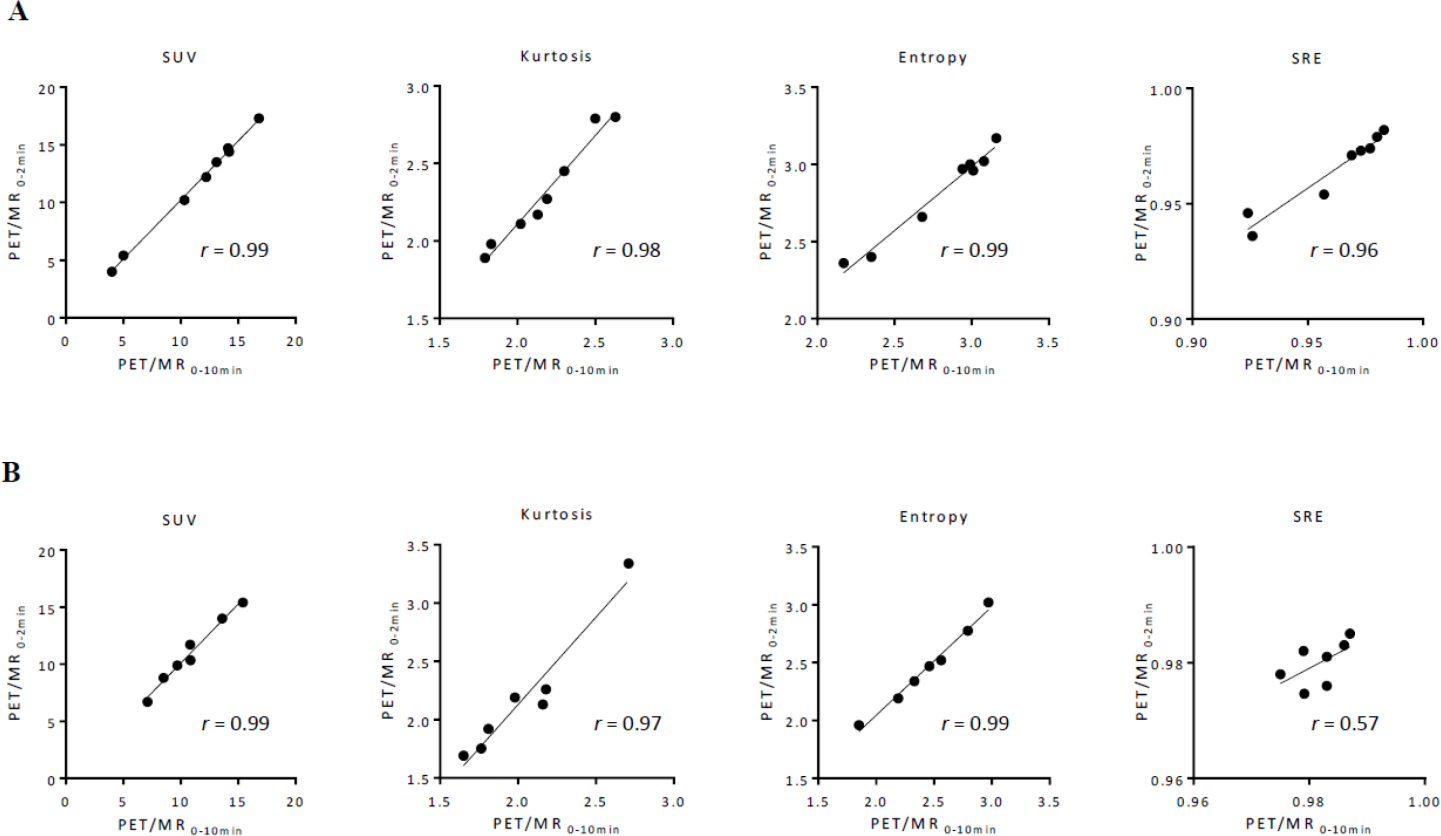
Relationships among features extracted using 64 bins (bin width = 0.4 SUV) between PET/MR_0–2 min_ and PET/MR_0–10 min_ images in gynecological cancer (**A**) and oral cavity/oropharyngeal cancer (**B**). A regression line is shown with Pearson’s correlation coefficient (*r*) and a *p* value.

Between PET/CT and PET/MR_0–2 min_ images, SUV, TMR, skewness, kurtosis, entropy, homogeneity, and SRE strongly correlated in gynecological cancer (*r* = 0.98, 0.97, 0.94, 0.90, 0.94, 0.96, and 0.88, respectively), while LRE moderately correlated (*r* = 0.58) (Figure 1A). In oral cavity/oropharyngeal cancer, SUV, TMR, skewness, kurtosis, entropy, and homogeneity strongly correlated between PET/CT and PET/MR_0–2 min_ images (*r* = 0.97, 0.98, 0.97, 0.97, 0.94, and 0.83, respectively), while SRE and LRE moderately correlated (*r* = 0.42 and 0.46, respectively) (Figure 1B). Between PET/MR_0–2 min_ and PET/MR_0-10 min_ images, SUV, TMR, skewness, kurtosis, entropy, homogeneity, SRE, and LRE strongly correlated in gynecological cancer (*r* = 0.99, 0.99, 0.99, 0.98, 0.99, 0.99, 0.96, and 0.89, respectively) (Figure 2A). In oral cavity/oropharyngeal cancer, SUV, TMR, skewness, kurtosis, entropy, and homogeneity strongly correlated between PET/MR_0–2 min_ and PET/MR_0–10 min_ images (*r* = 0.99, 0.99, 0.96, 0.97, 0.99, and 0.92, respectively), while SRE and LRE moderately correlated (*r* = 0.57 and 0.56, respectively) (Figure 2B).

### Comparison of PET radiomic features extracted using 64 bins (bin width = 0.4 SUV)

**Figure 3:**
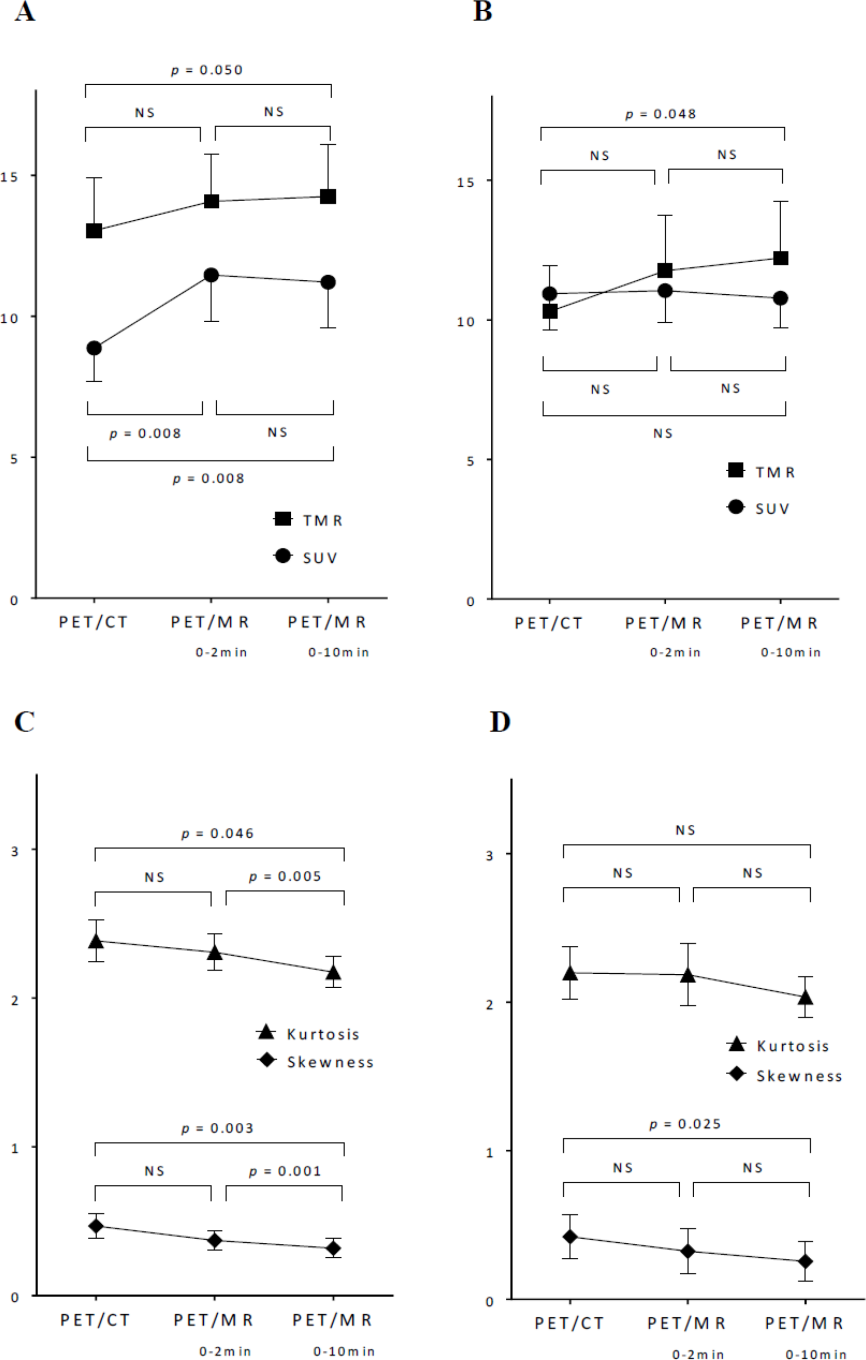
Comparisons of SUV, TMR, skewness, and kurtosis extracted using 64 bins (bin width = 0.4 SUV) among 3 images in gynecological cancer (**A**, **C**) and oral cavity/oropharyngeal cancer (**B**, **D**). Data represent the mean with error bars showing standard errors. The significance of differences was tested by a one-way repeated measures ANOVA. NS: not significant.

In the group analysis, SUVs on PET/MR_0–2 min_ and PET/MR_0–10 min_ were significantly higher than that on PET/CT in gynecological cancer (*p* = 0.008 and 0.008, respectively) (Figure 3A). On the other hand, no significant difference was observed between SUVs on PET/CT, PET/MR_0–2 min_, and PET/MR_0–10 min_ in oral cavity/oropharyngeal cancer (Figure 3B). In contrast, TMRs on PET/CT, PET/MR_0–2 min_, and PET/MR_0–10 min_ increased in this order in gynecological cancer and oral cavity/oropharyngeal cancer (Figures 3A and 3B).

Regarding shape-related histogram indices, skewness showed significant differences between PET/CT and PET/MR_0–10 min_, and between PET/MR_0–2 min_ and PET/MR_0–10 min_ in gynecological cancer (*p* = 0.003 and 0.001, respectively) (Figure 3C), and showed a significant difference between PET/CT and PET/MR_0–10 min_ in oral cavity/oropharyngeal cancer (*p* = 0.025) (Figure 3D). Kurtosis showed significant differences between PET/CT and PET/MR_0–10 min_, and between PET/MR_0–2 min_ and PET/MR_0–10 min_ (*p* = 0.046 and 0.005, respectively) (Figure 3C). Skewness and kurtosis on PET/CT, PET/MR_0–2 min_, and PET/MR_0–10 min_ decreased in this order in gynecological cancer and oral cavity/oropharyngeal cancer (Figure 3C and 3D).

**Figure 4:**
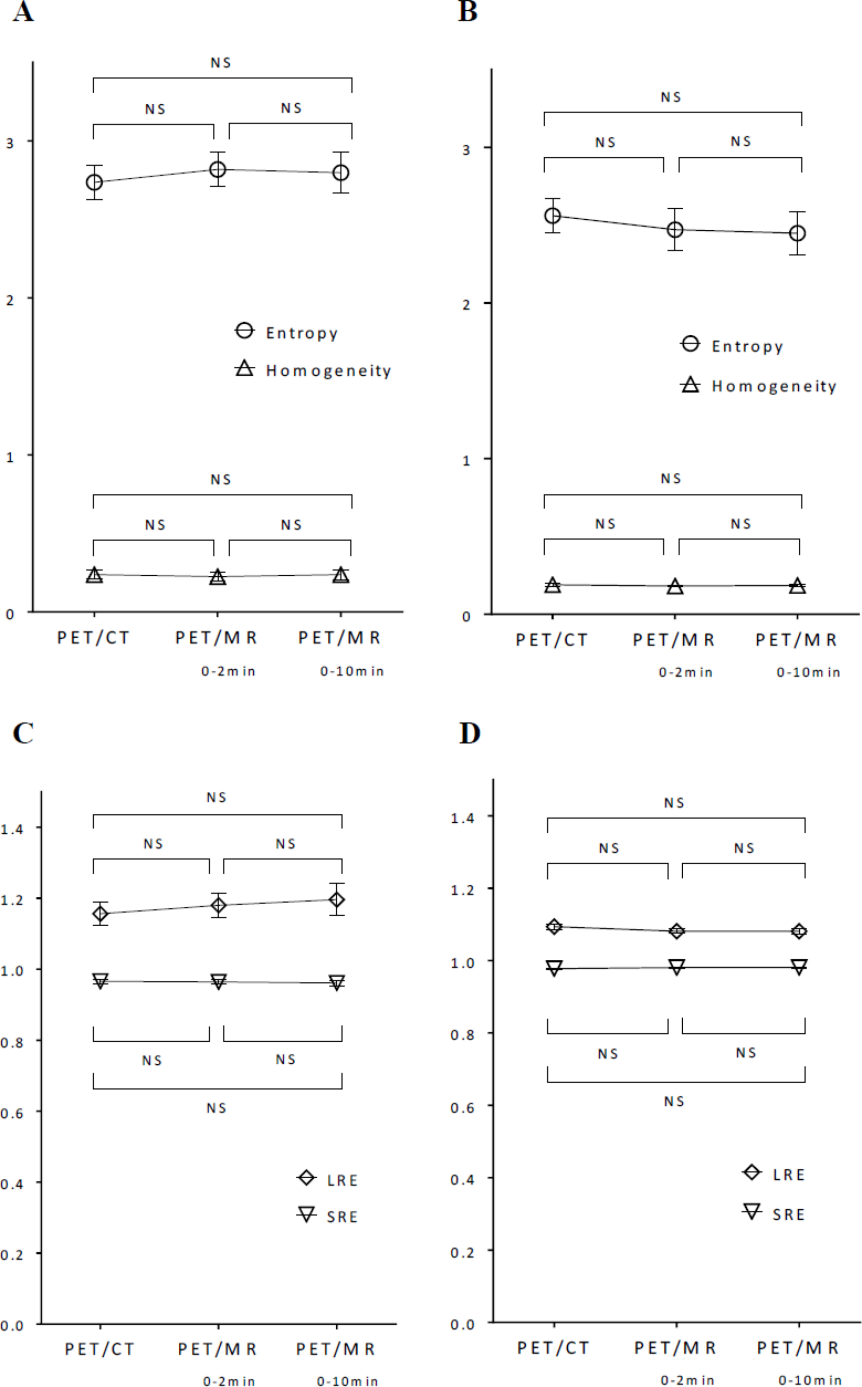
Comparisons of entropy, homogeneity, SRE, and LRE extracted using 64 bins (bin width = 0.4 SUV) among 3 images in gynecological cancer (**A**, **C**) and oral cavity/oropharyngeal cancer (**B**, **D**). Data represent the mean with error bars showing standard errors. The significance of differences was tested by a one-way repeated measures ANOVA. NS: not significant.

In contrast to conventional and histogram indices, 4 textural features (entropy, homogeneity, SRE, and LRE) extracted using 64 bins (bin width of 0.4 SUV) did not show any significant differences between PET/CT, PET/MR_0–2 min_, and PET/MR_0–10 min_ images in gynecological cancer and oral cavity/oropharyngeal cancer (Figure 4). Although PET/MR_0–10 min_ images were slightly more homogeneous visually than PET/MR_0–2 min_ images, PET textural features did not significantly differ between them.

### Impact of different bins on PET radiomic features

When different numbers of bins (8 and 256, equivalent to fixed bin widths of 3.1 and 0.1 SUV, respectively) were used, the conventional indices of SUV and TMR were unchanged and the histogram indices of skewness and kurtosis were mostly unchanged from those extracted using 64 bins (fixed bin width of 0.4 SUV) (Supplementary Figures 1 and 3). When the number of bins was changed from small (8 bins) to medium (64 bins) and large (256 bins), entropy increased, homogeneity decreased, and the difference in the measured values of SRE and LRE also decreased in this order (Supplementary Figures 2 and 4). Although 4 textural features extracted using 64 bins (bin width of 0.4 SUV) did not show any significant differences between 3 images, some textural features extracted using 8 and 256 bins showed significant differences between images (see Supplementary Figures 2 and 4).

### Representative cases

**Figure 5:**
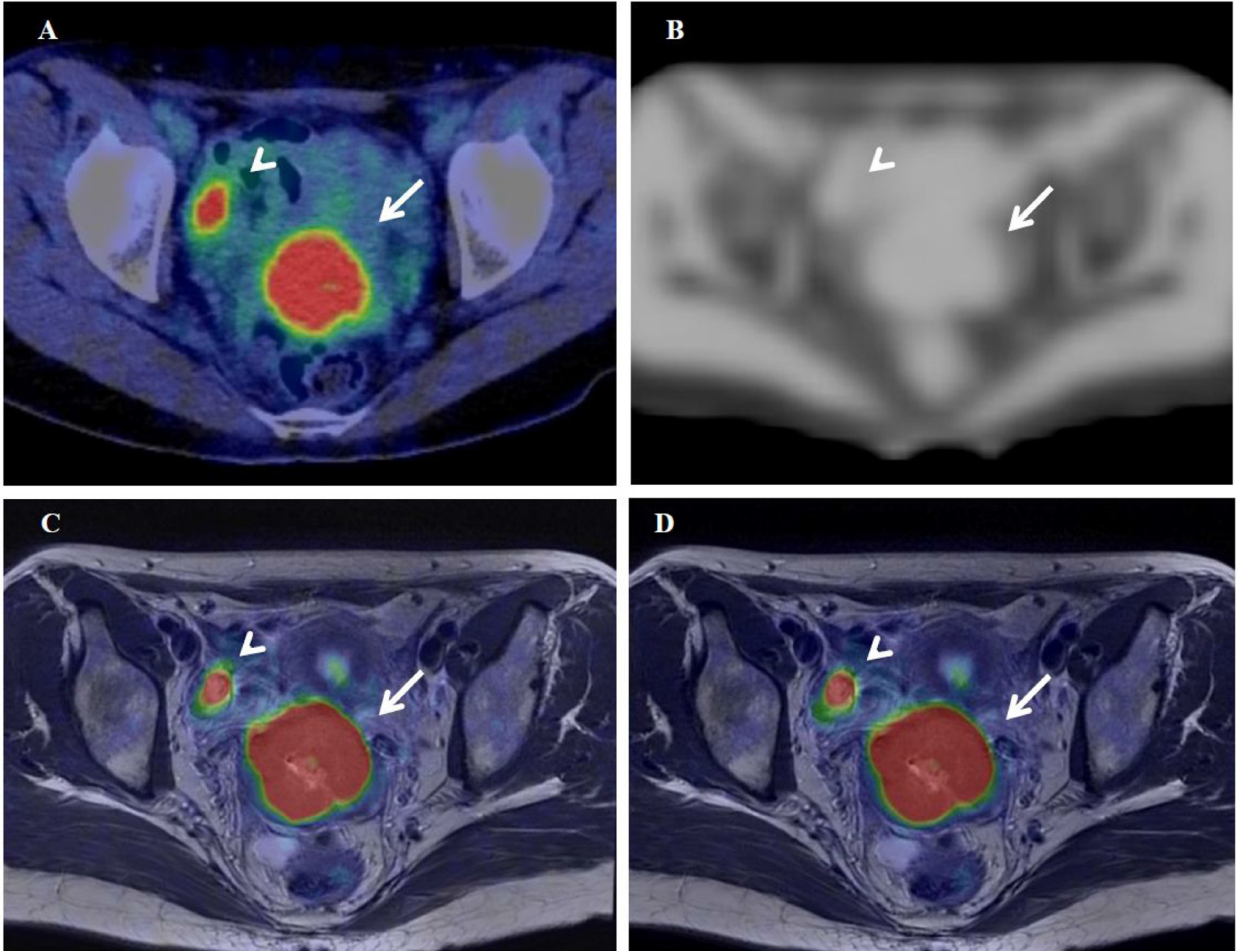
^18^F-FDG PET/CT (**A**), the MRAC map (**B**), and PET/MR_0-2min_- and PET/MR_0-10min_-T2WI fused images (**C** and **D**) of a 37-year-old woman with stage IB2 cervical cancer. Arrows and arrowheads show the primary tumor and normal right ovary, respectively.

**Figure 6:**
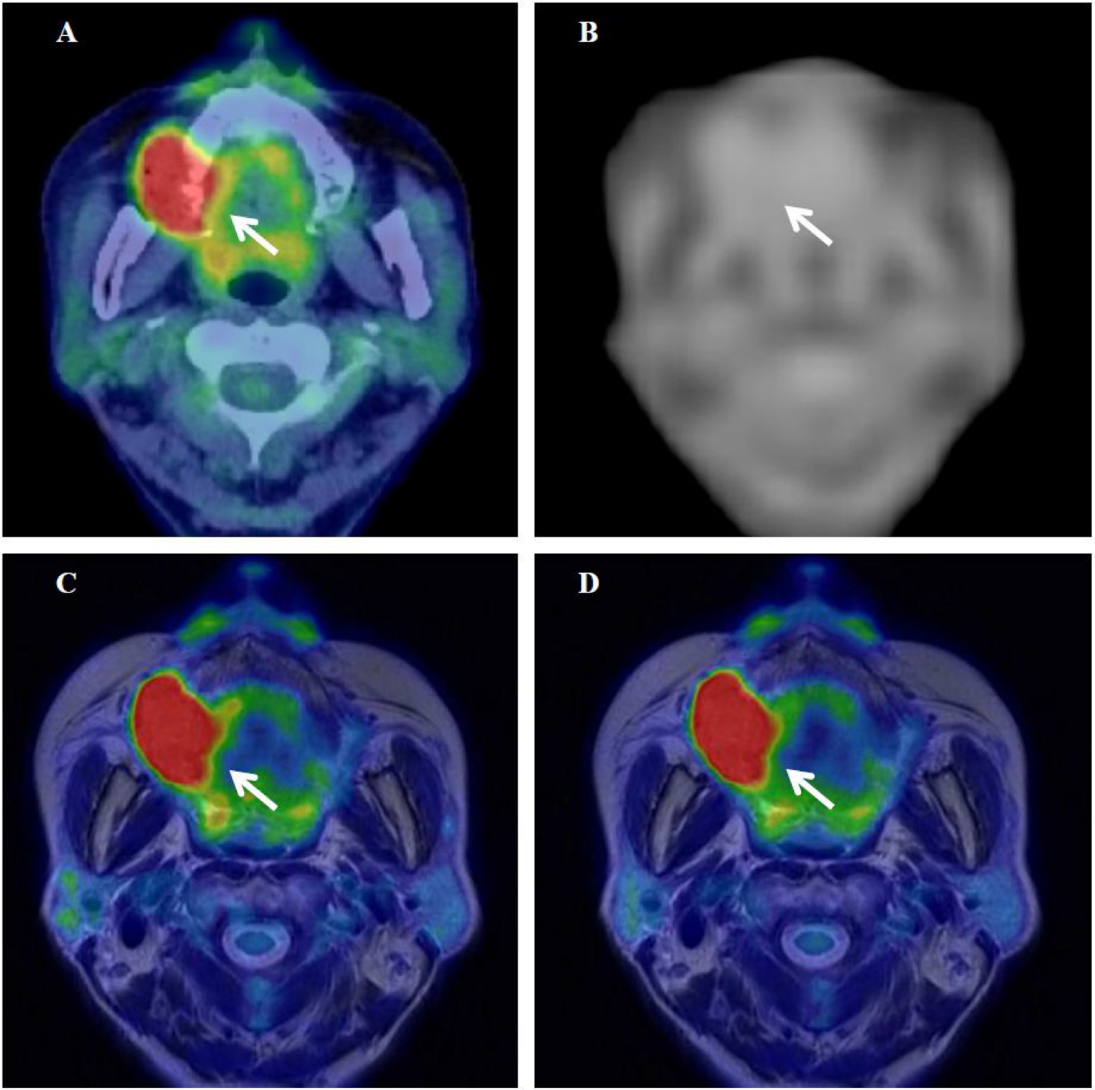
^18^F-FDG PET/CT (**A**), the MRAC map (**B**), and PET/MR_0-2min_- and PET/MR_0-10min_-T2WI fused images (**C** and **D**) of a 67-year-old woman with stage IVA upper gingival cancer. Arrows show the primary tumor.

Representative cases of cervical cancer and oropharyngeal cancer are shown in Figures 5 and 6, respectively. Textural features were extracted using 64 bins (fixed bin width of 0.4 SUV) in these cases.

Figure 5A–5D show ^18^F-FDG PET/CT, the MR-AC map, and PET/MR_0–2 min_- and PET/MR_0–10 min_-T2WI fused images of a 37-year-old woman with stage IB2 cervical cancer (#6 in Table 2), respectively. SUV of 14.4 on delayed PET/MR_0–2 min_ images was significantly higher (24%) than that of 11.6 on early PET/CT images. The Dixon-based MR-AC map (Figure 5B) did not assign the appropriate attenuation coefficient (μ value) to the pelvic bones, which may have resulted in insufficient AC underestimating tracer uptake on PET/MR images. However, SUV was markedly higher on PET/MR_0–2 min_ images than on PET/CT images in the cervical cancer patient, possibly because the biological factor (SUV increase in the primary tumor at a delayed scan period) was markedly larger than the underestimation of tracer uptake by MR-AC. The differences (|% difference|) observed in TMR, skewness, kurtosis, entropy, homogeneity, SRE, and LRE between PET/CT and PET/MR_0–2 min_ were 10%, 10%, 2%, 3%, 0%, 1%, and 7%, respectively.

**Table 2:** Patient characteristics

Department	Patient^#^	Age	Gender	Disease	Stage^*^
	1	67	F	Cervical cancer (SCC)	IIIB
	2	45	F	Cervical cancer (SCC)	IIIB
	3	67	F	Lt. fallopian tube cancer (HGSC)	IVB
Gynecology	4	40	F	Lt. ovarian cancer (CCA)	IC
	5	75	F	Endometrial cancer (SA)	IIIC2
	6	37	F	Cervical cancer (SCC)	IB2
	7	65	F	Cervical cancer (SCC)	IB1
	8	43	F	Cervical cancer (MA)	IIB
	9	66	M	Rt. oropharyngeal cancer (tongue base)	IVA
	10	68	F	Lt. lower gingival cancer	IVA
Otorhinolaryngology	11	87	F	Lt. oropharyngeal cancer (tonsil)	IVA
	12	67	F	Rt. upper gingival cancer	IVA
	13	58	M	Rt. oropharyngeal cancer	IVA
	14	81	M	Lt. hard plate cancer	III
	15	99	F	Rt. lower gingival cancer	IVA

SCC; squamous cell carcinoma, HGSC; high-grade serous carcinoma, CCA; clear cell adenocarcinoma,

SA; serous adenocarcinoma, MA; mucinous adenocarcinoma.

*Cancer staging is based on the International Federation of Gynecology and Obstetrics (FIGO) staging system in gynecology and on the American Joint Committee on Cancer (AJCC) TNM classification in otorhinolaryngology.

Figures 6A–6D show ^18^F-FDG PET/CT, the MR-AC map, and PET/MR_0–2 min_- and PET/MR_0–10 min_-T2WI fused images of a 67-year-old woman with stage IVA upper gingival cancer (#12 in Table 2), respectively. SUV of 14.0 on delayed PET/MR_0–2 min_ images was slightly higher (2%) than that of 13.7 on early PET/CT images in this patient. The MR-AC map (Figure 6B) did not assign the appropriate μ value to the jaw bones, which may have resulted in insufficient AC underestimating tracer uptake on PET/MR images. SUV on PET/MR_0–2 min_ images was similar to that on PET/CT images in the upper gingival cancer patient, possibly because the biological effect at the delayed scan period was canceled out by the gross underestimation of tracer uptake by MR-AC. The differences (|% difference|) observed in TMR, skewness, kurtosis, entropy, homogeneity, SRE, and LRE between PET/CT and PET/MR_0–2 min_ were 29%, 63%, 14%, 7%, 10%, 0%, and 1%, respectively.

## DISCUSSION

This is an initial study that evaluated the impact of differences in the scanner, scan protocol, and lesion location on ^18^F-FDG PET radiomic features using the latest PET/CT and PET/MR scanners in patients. ^18^F-FDG PET radiomic features strongly correlated between PET/CT and PET/MR images despite the difference between CT-AC and MR-AC. In group comparisons, SUVs were significantly higher on PET/MR_0–2 min_ and PET/MR_0–10 min_ images (delayed scans) than on PET/CT (early scan) in gynecological cancer, possibly because the biological factor (SUV increase in the primary tumor at a delayed scan time) was markedly larger than the underestimation of tracer uptake by MR-AC errors of pelvic bones. On the other hand, SUVs on PET/MR images were similar to those on PET/CT in oral cavity/oropharyngeal cancer, possibly because the biological effects in the longer waiting period of the PET/MR scan were canceled out by the gross underestimation of tracer uptake by MR-AC errors of the jaw bones. In other words, MR-AC of the jaw bones underestimated tumor tracer uptake in oral cavity/oropharyngeal cancer more significantly than that of the pelvic bones in gynecological cancer. These results are consistent with previous findings by Samarin *et al.* showing the prominent underestimation of tracer uptake in bone lesions providing whole-body error maps [18]. Our study included 3 gingival cancers and one hard plate cancer in the oral cavity/oropharyngeal cancer group (Table 2 and Figure 6), and these oral cavity lesions close to the jaw bones may have been affected more by MR-AC errors than gynecological tumors at a short distance from the pelvic bones. New MR-AC methods including bone information such as model-based and fast zero-echo-time (ZTE)-based MR-AC are currently being developed for whole-body scans [19, 20].

The selected textural features (entropy, homogeneity, SRE, and LRE) extracted using the medium number of bins of 64 were more stable parameters than SUV and other histogram indices between 3 different images in the present study. The stability of textural features between images may be due to the resampling process prior to calculating textural features. In the resampling process, voxel intensities are discretized into a reduced number of discrete values (Eq. 2). The discretization of voxel intensities may reduce the difference in tracer uptake between scanners and scan protocols. The stability of textural features extracted using a medium number of bins (64 in this study) has advantages and disadvantages for textural features to be used as quantitative measures in the field of medical imaging. The advantage is that the stability of textural features enhances multicenter studies in which different scanners and scan protocols are used at different sites. On the basis that voxel sizes and reconstruction parameters are adjusted, selected textural features may be gathered from multiple institutes and evaluated with patient information including the tumor phenotype, genotype, treatment response, and long-term prognosis. This large amount of data will lead to precise diagnoses, potential prognostic models, and effective therapeutic strategies using radiogenomic approaches [21, 22]. The disadvantage is that it appears to be difficult to express a subtle difference in the homogenous nature between images using textural features. In the present study, shape-related histogram indices (skewness and kurtosis) on PET/CT, PET/MR_0–2 min_, and PET/MR_0–10 min_ decreased in this order and showed significant differences between images in gynecological cancer and oral cavity/oropharyngeal cancer, namely, longer waiting periods and a longer scan duration provide a more uniformly distributed (symmetric and platykurtic) histogram of tracer uptake. Although PET/MR_0–10 min_ images were slightly more homogeneous visually than PET/MR_0–2 min_ images in the present study (Figures 5 and 6), PET textural features were not significantly different. Entropy extracted using 64 bins slightly decreased and homogeneity slightly increased from PET/MR_0–2 min_ to PET/MR_0–10 min_ images (Figures 4A and 4B).

In order to reduce the influence of the tumor size on textural feature measurements in the present study, absolute resampling methods with fixed bin widths of 0.1, 0.4, and 3.1 SUV units (256, 64, and 8 bins, respectively) were used instead of relative resampling methods, introducing a large bias associated with the tumor volume [23]. When using the large number of bins of 256, equivalent to a bin width of 0.1 SUV which is almost equal to un-discrete SUV unit, the stability of entropy between PET/CT, PET/MR_0–2 min_ and PET/MR_0–10 min_ images nearly disappeared and the values of entropy increased in this order in gynecological cancer (Supplementary Figure 4). Although the difference in entropy between PET/MR_0–2 min_ and PET/MR_0–10 min_ images was not significant, higher entropy on PET/MR_0–10 min_ than PET/MR_0–2 min_ was inconsistent with visual impressions. Eight bins appeared to be too small to extract robust textural features because the rough discretization of PET images by the bin width of 3.1 SUV resulted in larger standard errors in entropy and LRE than 64 bins (0.4 SUV) (Supplementary Figure 2). The medium bin width may be appropriate for texture feature extraction in oncology, and the optimal bin width needs to be investigated according to tumor type. Since the discretization method may have an important impact on the resulting texture features [24], further validations with a larger patient population and different types of cancers are required.

There are some limitations in the present study. The scan duration per 1 bed position and voxel size were not perfectly matched between PET/CT and PET/MR_0–2 min_ images. Scan durations were 1.8 min/bed in PET/CT and 2 min/bed in PET/MR_0–2 min_ images. Voxel sizes were 4 × 4 × 2 mm in PET/CT and 4 × 4 × 2.78 mm in PET/MR images. These differences may have affected the results obtained for histogram and textural feature quantification in the present study. Since PET textural features are dependent on various factors such as image acquisition, reconstruction, preprocessing, segmentation, and mathematical methods [24], the standardization of a PET texture analysis will be necessary for inter-institutional evaluations in the future.

In summary, ^18^F-FDG PET radiomic features strongly correlated between PET/CT and PET/MR images. Dixon-based AC on PET/MR images underestimated tumor tracer uptake more significantly in oral cavity/oropharyngeal cancer than in gynecological cancer. ^18^F-FDG PET textural features extracted using a medium number of bins were affected less by differences in the scanner and scan protocol than conventional and histogram features. The stability of textural features enhances multicenter studies in which different scanners and scan protocols are used at different sites.

## MATERIALS AND METHODS

### Study design and patients

**Figure 7:**
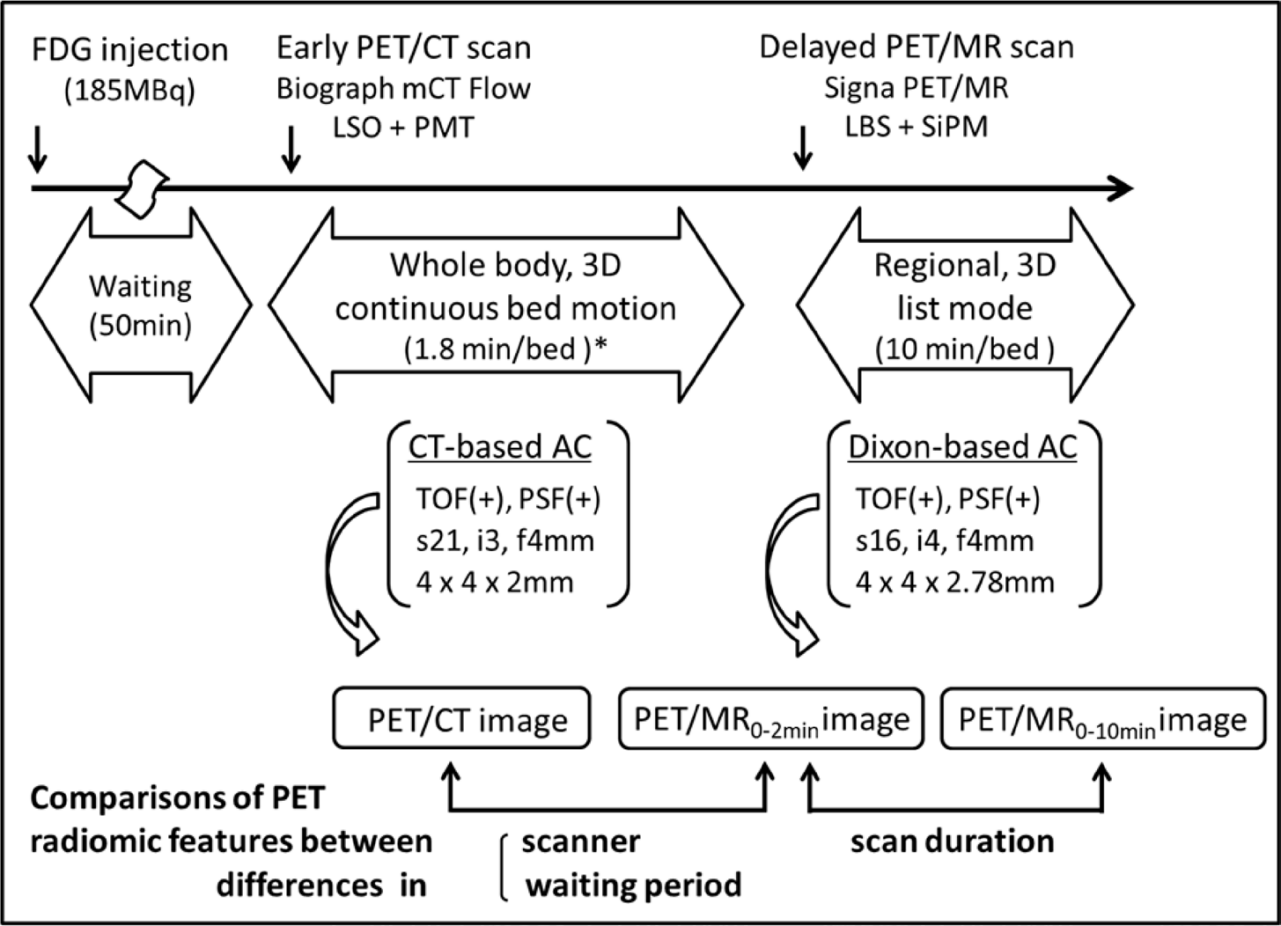
Study outline map ^*^A bed speed of 1.2 mm/s, equivalent to 1.8 min/bed in the step acquisition mode.

The outline of the present study is shown in Figure 7. In this retrospective analysis, we evaluated 8 consecutive patients with a histological diagnosis of gynecological cancer (mean age = 54.9 ± 15.0 years) between November 2015 and May 2016, and 7 consecutive patients with a histological diagnosis of oral cavity/oropharyngeal cancer (mean age = 75.1 ± 14.4 years) between April 2017 and August 2017. Patient characteristics are summarized in Table 2. They underwent a whole-body ^18^F-FDG PET/CT scan (early scan) for staging and then a regional ^18^F-FDG PET/MR scan (delayed scan) for further evaluations on the same day at the University of Fukui Hospital. All patients provided oral consent to undergo a subsequent PET/MR scan with a safety check sheet for the MR examination after the PET/CT scan. This study protocol was approved by the Ethics Committee of the Faculty of Medical Sciences, University of Fukui, and the requirement to obtain formal written consent was waived.

### PET/CT scanner and scan protocol

A whole-body PET/CT scan was performed with Biograph mCT Flow (Siemens Healthcare, Erlangen, Germany), consisting of a PET detector with 4 rings, 48 detector blocks in each ring, and lutetium oxyorthosilicate (LSO) crystals of 4 × 4 × 20 mm in a 13 **×** 13 array coupled to a 2 × 2 photomultiplier tube (PMT) array in each detector block. The detector ring diameter was 84.2 cm, covering a transaxial PET field of view (FOV) of 70 cm and axial PET FOV of 21.6 cm. The time coincidence window was 4.1 ns, system time resolution 540 ps, and energy window 435–650 keV. All patients fasted for at least 4 hours prior to an intravenous injection of 185MBq ^18^F-FDG. Fifty min after the injection, patients were positioned supine in the PET/CT scanner, and low-dose helical CT (120 keV, 25 mAs, 512 × 512 matrix) in shallow breathing was performed for CT-AC. After the CT transmission scan, a whole-body PET scan was performed from the head to the inguinal region in the 3D acquisition and continuous bed motion (CBM) mode with a bed speed of 1.2 mm/s, which is equivalent to 1.8 min/bed in the step acquisition mode. Whole-body PET data were reconstructed with TOF, PSF, a standard iterative algorithm (ordered subset expectation maximization [OSEM] selecting 21 subsets and 3 iterations), and post-smoothing with a 4-mm Gaussian filter. The whole body image matrix size was 200 × 200 with 4 × 4 × 2 mm voxels. Reconstructed images (PET/CT images) were then converted to semiquantitative images corrected by the injected dose and subject’s body weight (=SUV).

### PET/MR scanner and scan protocol

After the whole-body PET/CT scan, patients were transferred to the TOF PET/MR scanner: Signa PET/MR (GE Healthcare, Waukesha, WI, USA), consisting of a PET detector with 5 rings, 28 detector modules × 5 units × 4 blocks in each ring, and lutetium-based scintillator (LBS) crystals of 4 × 5.3 × 25 mm in a 4 × 9 array coupled to a 1 × 3 SiPM array in each detector block. The detector ring diameter was 62 cm, covering a transaxial PET FOV of 60 cm and axial PET FOV of 25 cm. The time coincidence window was 4.57 ns, system time resolution <400 ps, and energy window 425–650 keV. Regarding MR-AC, a 2-point Dixon 3D T1-weighted fast SPGR sequence (TR/TE1/TE2: 4.0/1.1/2.2 ms; FOV 50 × 37.5 cm; matrix 256 × 128; slice thickness/overlap: 5.2/2.6 mm; 120 image/slab; imaging time: 18 sec) was acquired. Dixon-based MR-AC recognizes body tissues as soft tissue, fat, and air. The regional PET scan was performed in the 3D acquisition and list mode with a 10 min/bed position (89 slices/bed) in 1–2 beds with a 24-slice overlap. The regional PET data of 0–2 min and 0–10 min were separately reconstructed with TOF, PSF, OSEM selecting 16 subsets and 4 iterations, and post-smoothing with a 4-mm Gaussian filter. The regional image matrix size was 128 × 128 with 4 × 4 × 2.78 mm voxels. Reconstructed images (PET/MR_0–2 min_ and PET/MR_0–10 min_ images) were then converted to SUV images.

### PET radiomic feature measurement

The entire radiomic feature extraction was performed using LIFEx software version 3.12 (Local Image Feature Extraction, www.lifexsoft.org) [25]. The primary tumor of each patient was delineated on PET/CT, PET/MR_0–2 min_ and PET/MR_0–10 min_ images using an adaptive threshold method (contrast-oriented algorithm) as described [26, 27]:

$$T=\beta \times I_{\mathrm{70}}+I_{bgd}$$ Eq.1

where *T* is the threshold value and β = 0.3 was optimized by measurements in a Jaszczak phantom [28]. *I*_*70*_ was the mean uptake in a contour containing all voxels with a value greater than 70% of the maximum uptake in the tumor. *I*_*bgd*_ was defined as the mean uptake in a shell with a thickness of 2 voxels and located 6 voxels from the region used to calculate *I*_*70*_, and only voxels with uptake less than 2.5 SUV units were included in the calculation of *I*_*bgd*_. The volume of interest (VOI) on the primary tumor was recorded and used in a subsequent analysis.

Prior to radiomic feature computation, VOI voxel intensities were resampled using the absolute resampling method with fixed bounds and 64 discrete values [23, 29, 30]:

$$R\left(x\right)=round\left[64\times \frac{I\left(x\right)-lower\;bound}{upper\;bound-lower\;bound}\right]$$ Eq.2

where *R(x)* is voxel intensity after discretization and *I(x)* is that before discretization. The lower bound was set to 0 and the upper bound was set to 25 SUV units, which corresponded to the maximum intensity over all of the primary tumors included in this study. As a result, a sampling bin width of 0.4 SUV units was used. In order to examine the impact of different numbers of bins (i.e. bin widths), 8 and 256 bins, equivalent to fixed bin widths of 3.1 and 0.1 SUV units, respectively, were used in additional analyses. The SUV histogram, grey-level co-occurrence matrix (GLCM), and gray-level run length matrix (GLRLM) were used in assessments of first-, second-, and high-order radiomic features, respectively. Seven indices were extracted: average SUV (SUV) as a conventional feature, skewness and kurtosis as histogram features (shapes of distributions), entropy and homogeneity from GLCM, and short-run emphasis (SRE) and long-run emphasis (LRE) from GLRLM as textural features. The 4 textural features were selected due to their previously demonstrated robustness with respect to the segmentation method in each texture correlation group [27]. In addition, the tumor-to-muscle SUV ratio (TMR) was calculated using the gluteal muscles in gynecological cancer and the posterior neck muscles in oral cavity/oropharyngeal cancer. As a result, 8 PET radiomic features for each number of bins (8, 64, and 256) were evaluated in the present study (Table 1).

### Statistical analysis

Data are given as the mean ± SD. All statistical analyses were performed using SPSS statistics version 22, and *p* < 0.05 was considered to be significant. Regression analyses of radiomic features between PET/CT, PET/MR_0–2 min_, and PET/MR_0–10 min_ images were performed using Pearson’s correlation coefficient (*r*). Differences in radiomic features between the three images were assessed using a one-way repeated measures analysis of variance (ANOVA).

## SUPPLEMENTARY MATERIALS FIGURES


