# Supplementary material for: Selected PET radiomic features remain the same
Source: Oncotarget. 2018 Apr 17;9(29):20734–46. doi: 10.18632/oncotarget.25070 (PMC5945508; doi:10.18632/oncotarget.25070)
Supplement: Supplementary file 1 [file oncotarget-09-20734-s001.pdf]

Selected PET radiomic features remain the same

SUPPLEMENTARY MATERIALS

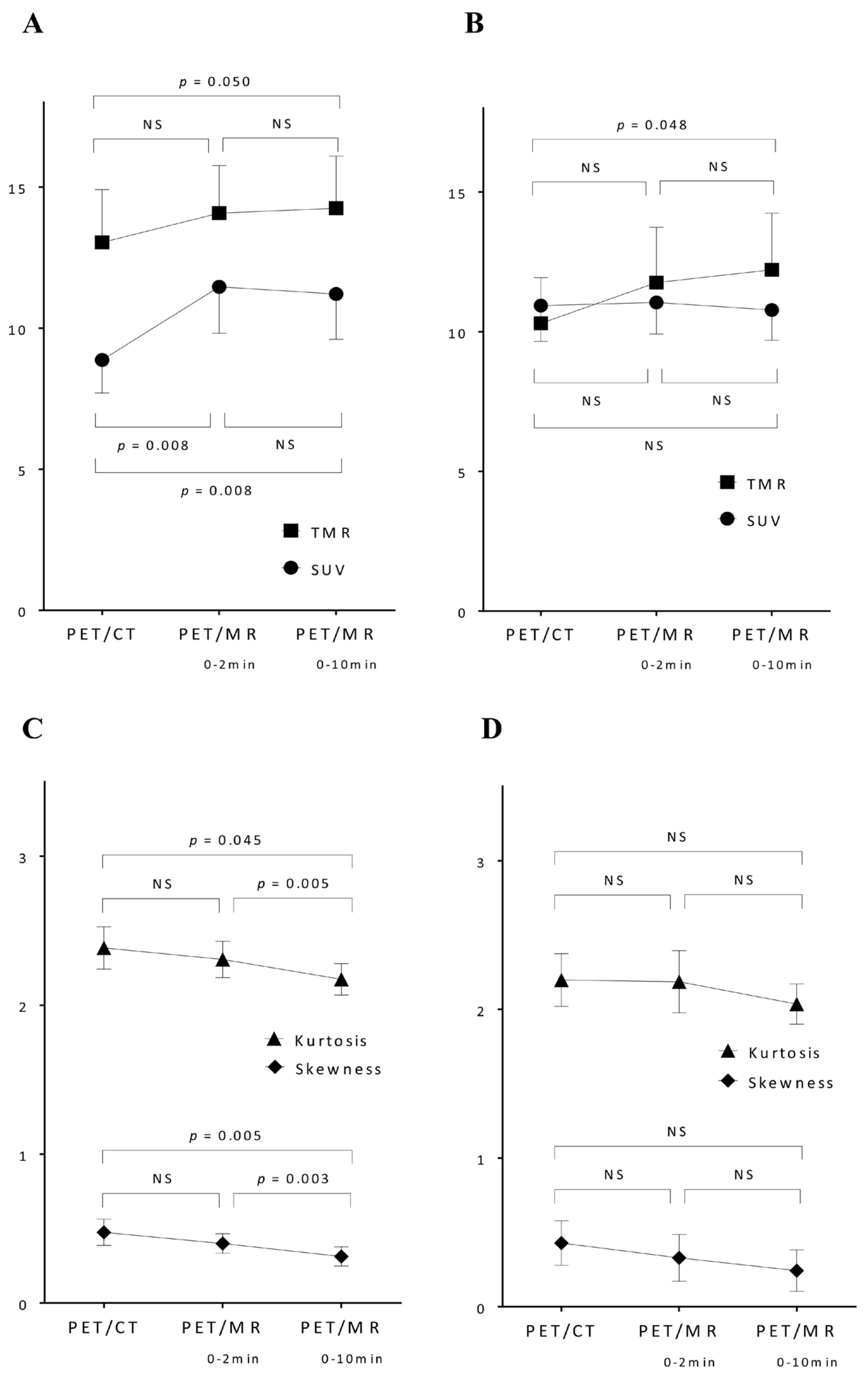

Supplementary Figure 1: Comparisons of SUV, TMR, skewness, and kurtosis extracted using 8 bins (bin width = 3.1 SUV) among 3 images in gynecological cancer (**A**, **C**) and oral cavity/oropharyngeal cancer (**B**, **D**). Data represent the mean with error bars showing standard errors. The significance of differences was tested by a one-way repeated measures ANOVA. NS: not significant.

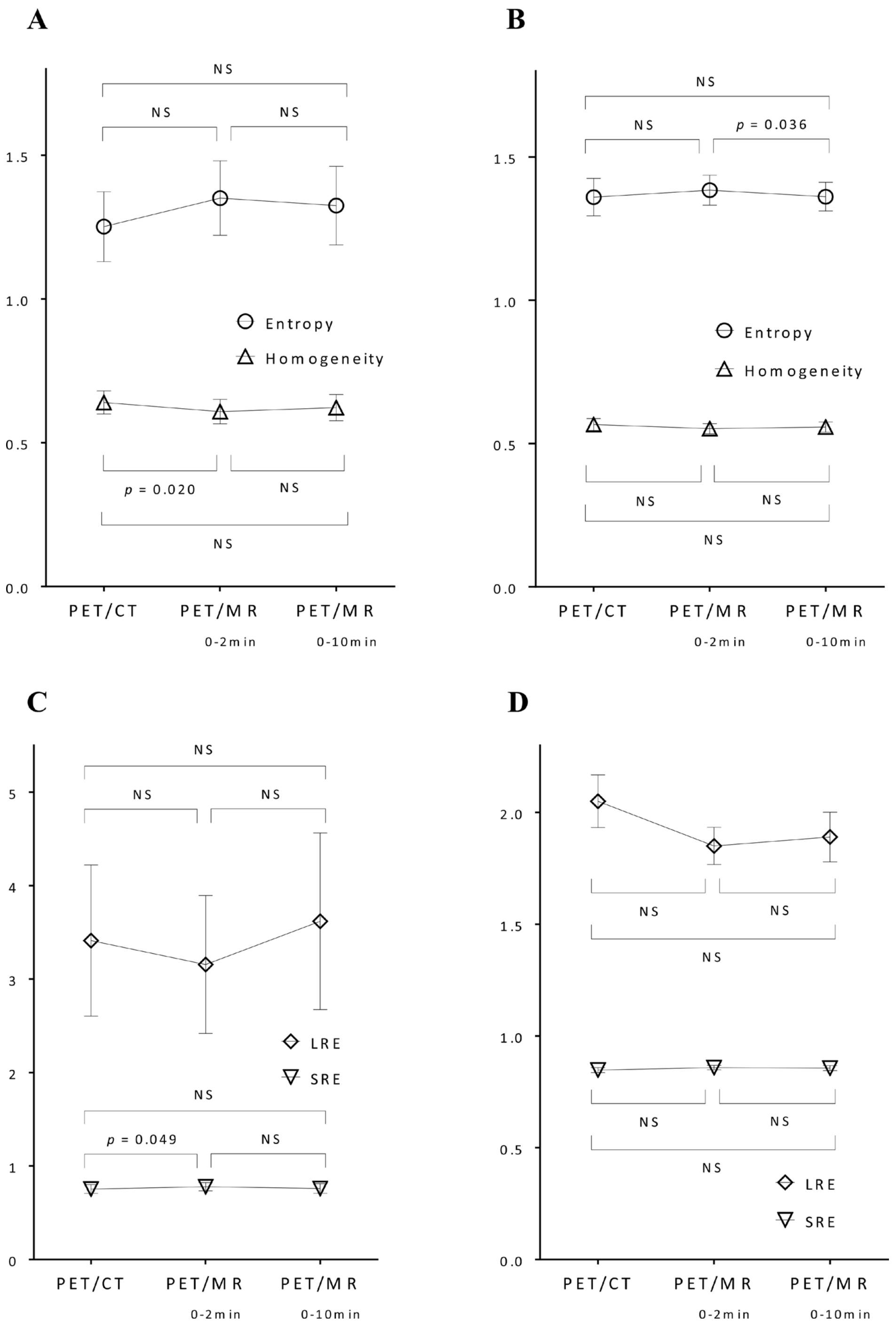

Supplementary Figure 2: Comparisons of entropy, homogeneity, SRE, and LRE extracted using 8 bins (bin width = 3.1 SUV) among 3 images in gynecological cancer (**A**, **C**) and oral cavity/oropharyngeal cancer (**B**, **D**). Data represent the mean with error bars showing standard errors. The significance of differences was tested by a one-way repeated measures ANOVA. NS: not significant.

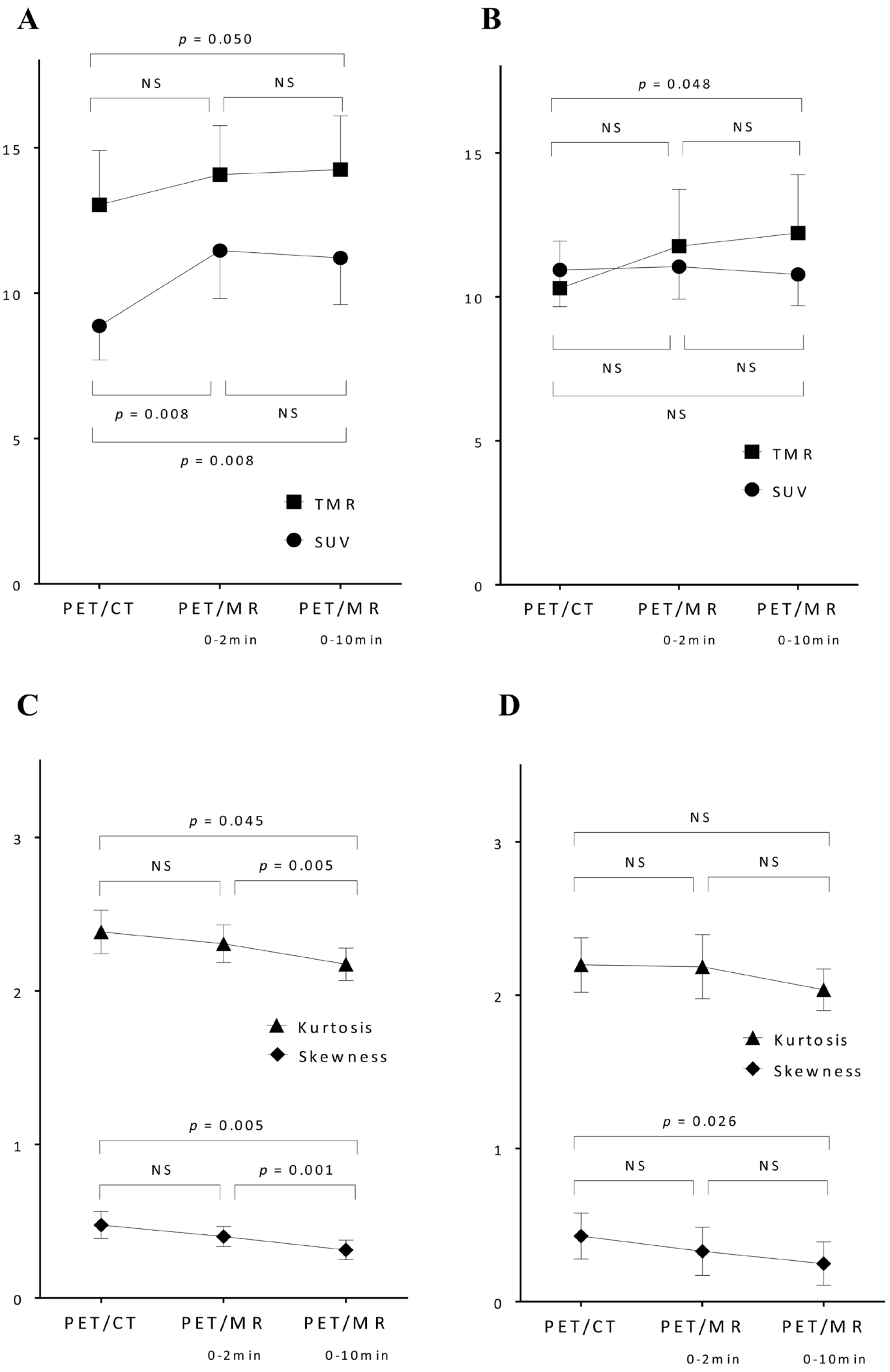

Supplementary Figure 3: Comparisons of SUV, TMR, skewness, and kurtosis extracted using 256 bins (bin width = 0.1 SUV) among 3 images in gynecological cancer (**A**, **C**) and oral cavity/oropharyngeal cancer (**B**, **D**). Data represent the mean with error bars showing standard errors. The significance of differences was tested by a one-way repeated measures ANOVA. NS: not significant.

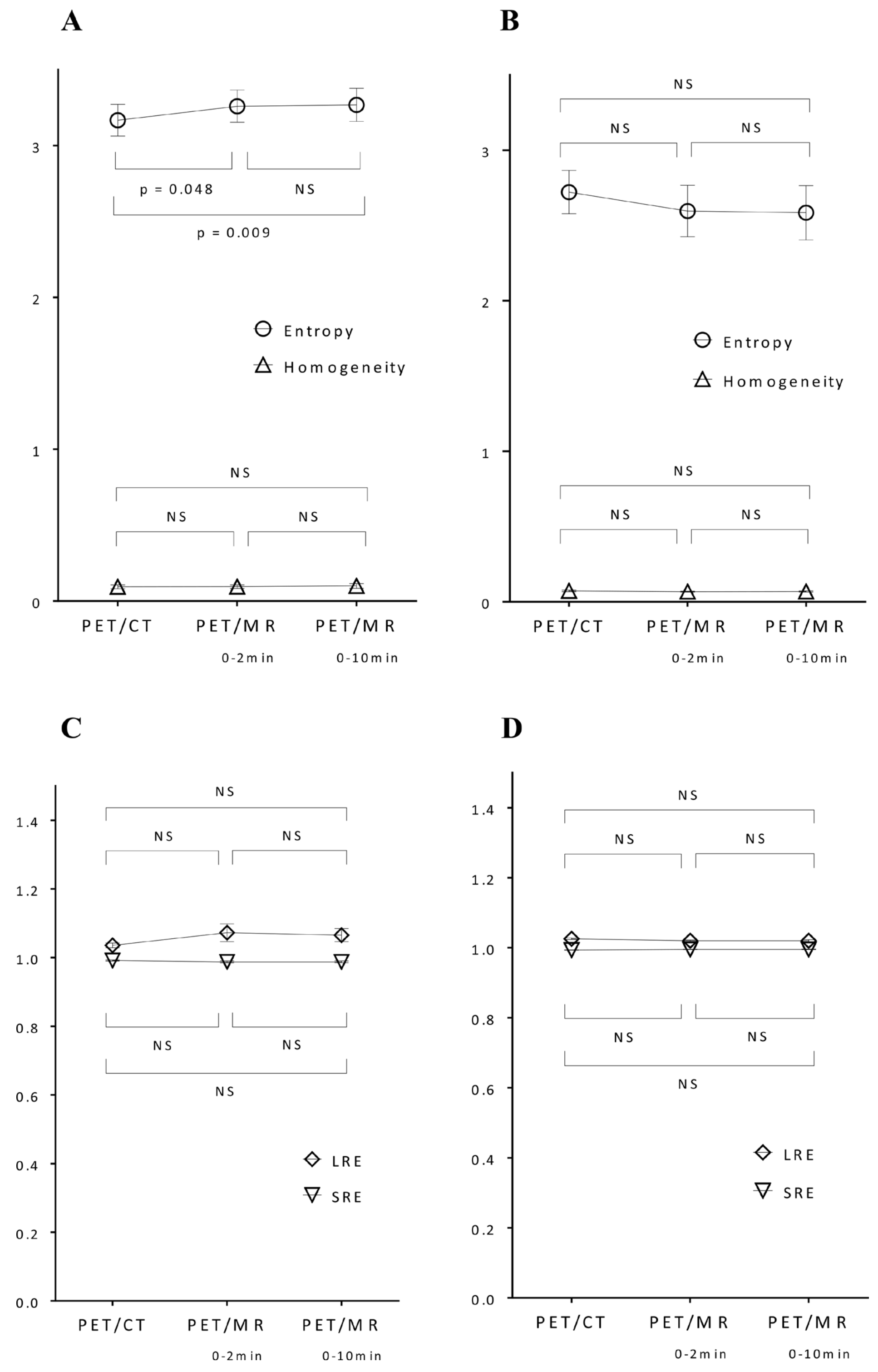

Supplementary Figure 4: Comparisons of entropy, homogeneity, SRE, and LRE extracted using 256 bins (bin width = 0.1 SUV) among 3 images in gynecological cancer (**A**, **C**) and oral cavity/oropharyngeal cancer (**B**, **D**). Data represent the mean with error bars showing standard errors. The significance of differences was tested by a one-way repeated measures ANOVA. NS: not significant.
